# Cancer-control outcomes with [^177^Lu]Lu-PSMA Radioligand Therapy in elderly, frail or comorbid mCRPC patients

**DOI:** 10.7150/thno.108963

**Published:** 2025-01-27

**Authors:** Mike Wenzel, Carolin Siech, Cristina Cano Garcia, Clara Humke, Daniel Groener, Maximilian Kriegmair, Markus Graefen, Tobias Maurer, Georg Salomon, Severine Banek, Felix K. H. Chun, Philipp Mandel

**Affiliations:** 1 Department of Urology, University Hospital Frankfurt, Goethe University Frankfurt am Main, Frankfurt, Germany.; 2 Department of Nuclear Medicine, University Hospital Frankfurt, Goethe University Frankfurt am Main, Frankfurt, Germany.; 3 Urologische Klinik München Planegg, Planegg, Germany.; 4 Department of Urology and Urosurgery, University Medical Center Mannheim, Medical Faculty Mannheim, Heidelberg University, Mannheim, Germany.; 5 Martini-Klinik Prostate Cancer Center, University Hospital Hamburg-Eppendorf, Hamburg, Germany.; 6 Department of Urology, University Hospital Hamburg-Eppendorf, Hamburg, Germany.

**Keywords:** mCRPC, PFS, OS, Survival, Lu-177

## Abstract

**Rationale:** [^177^Lu]Lutetium prostate-specific membrane antigen radioligand therapy ([^177^Lu]Lu-PSMA) is EMA-approved for certain indications in metastatic castration resistant prostate cancer (mCRPC). However, cancer-control outcomes in specific and trial-underrepresented subgroups are scant.

**Methods:** We relied on the FRAMCAP database to elaborate progression-free (PFS) and overall (OS) survival in elderly (≥75 yrs), frail (ECOG status ≥1) mCRPC patients or those with cardiovascular disease (CVD) treated with [^177^Lu]Lu-PSMA.

**Results:** Of 312 [^177^Lu]Lu-PSMA mCRPC patients, 76% were ≤75 vs. 24% >75 years. Patients >75 years received [^177^Lu]Lu-PSMA more frequently within the first three mCRPC lines (85% vs. 62%) and harbored more frequently ECOG status ≥2 (13% vs. 4.3%, both p < 0.01). In PFS and OS analyses, no significant difference between patients aged ≤75 vs. >75 years was observed (hazard ratios [HR] 0.97 & 0.85, both p≥0.4) with median PFS of 12.7 vs. 11.7 and OS of 15.1 vs. 19.8 months. In ECOG-stratified analyses, no PFS difference was observed, with significantly better OS for ECOG 0 vs. ≥1 (HR 1.69, p < 0.01), but not after further multivariable adjustment. In CVD-stratified analyses, PFS failed to provide significant differences between CVD vs. no CVD (HR: 1.44, p = 0.051). However, in OS analyses, significant worse OS for CVD mCRPC [^177^Lu]Lu-PSMA patients was observed (HR: 1.93, p < 0.01). After multivariable adjustment, CVD was an independent predictor for worse PFS and OS (both p < 0.01).

**Conclusions:** Real-world evidence suggests equally effective cancer-control outcomes in elderly and frail mCRPC patients treated with [^177^Lu]Lu-PSMA. However, patients with CVD are of higher risk for shorter PFS and OS.

## Introduction

Patients with metastatic castration-resistant prostate cancer (mCRPC) face a high risk of cancer-specific mortality and significant disease-related complications due to tumor burden 1. In recent years, the landscape of mCRPC treatment significantly changed and several new systemic treatment options and combinations have been approved, by demonstrating improvements in progression-free survival (PFS) and overall survival (OS) 2-10. Moreover, recently the European Medicines Agency (EMA) approved [^177^Lu]Lutetium-vipivotidtetraxetan prostate-specific membrane antigen radioligand therapy ([^177^Lu]Lu-PSMA) for mCRPC patients previously treated with androgen receptor pathway inhibitors (ARPI) and taxane-based chemotherapy, based on the results of the VISION trial 11. [^177^Lu]Lu-PSMA therapy, which targets prostate cancer cells with beta radiation via a molecular approach distinct from ARPI or taxane-based chemotherapy, has emerged as a pivotal component in the sequential treatment strategies for mCRPC due to its cancer-control efficacy. These effects have been further elaborated in additional randomized phase III trials in earlier treatment lines and stages of mCRPC or also in combination with ARPI administration 12-15.

Currently, elderly mCRPC patients or those with severe comorbidities or advanced frailty index such as Eastern Cooperative Oncology Group (ECOG) status are often underrepresented within phase III trials. Nonetheless, several post-hoc analyses of phase III trials in metastatic prostate cancer (hormone-sensitive [mHSPC] or mCRPC) suggested different and mostly worse cancer-control outcomes such as PFS and OS in specific patient subgroups, such as elderly or frail patients 11,16,17. However, no data on these specific mCRPC patient subgroups treated with [^177^Lu]Lu-PSMA are currently available.

We addressed this knowledge gap and relied on the Frankfurt Metastatic Cancer Database of the Prostate (FRAMCAP) to elaborate cancer-control outcomes such as PFS and OS in [^177^Lu]Lu-PSMA-treated mCRPC patients. We hypothesized important cancer-control differences may exist in mCRPC patients treated with [^177^Lu]Lu-PSMA above 75 years, ECOG performance status ≥1 or with severe comorbidities such as history or active cardiovascular disease (CVD).

## Materials and Methods

### Study population

After receiving approval from the local ethics committee (reference number: SUG-5-2024) and adhering to the principles of the Declaration of Helsinki, we performed a retrospective analysis of all mCRPC patients documented in the prospectively maintained FRAMCAP database. A total of 1,182 patients treated at the Department of Urology, University Hospital Frankfurt, Germany, were screened. For the analysis, mCRPC patients were included if they had received at least one cycle of [^177^Lu]Lu-PSMA. This selection criteria resulted in 312 eligible mCRPC patients.

### [^177^Lu]Lu-PSMA radioligand therapy

Treatment of [^177^Lu]Lu-PSMA was administered at the nuclear medicine department every 6-8 weeks, as previously described 18-20. [^177^Lu]Lu-PSMA could be administered in accordance with EMA approval or as an individual compassionate use after previous multidisciplinary team discussion (MTD). For [^177^Lu]Lu-PSMA therapy, PSMA-PET/CT scan confirming PSMA-avid metastatic disease was required before initiating treatment.

### Statistical analysis

Descriptive statistics included the computation of frequencies and proportions for categorical variables used in the analysis. Median values and interquartile ranges (IQR) were reported for continuous variables. Statistical significance for differences in proportions was assessed using the Chi-square test, while the t-test and Kruskal-Wallis test were employed to evaluate differences in distributions.

For PFS and OS analyses, Kaplan-Meier curve estimates with a log-rank test were used. PFS was defined as the time from beginning of [^177^Lu]Lu-PSMA until beginning of next sequential treatment administration for mCRPC. OS was calculated from the start of [^177^Lu]Lu-PSMA radioligand therapy. We conducted three different sets of PFS and OS analyses: First, patients aged >75 years at metastatic disease vs. ≤75 years, second ECOG status 0 at metastatic disease vs. ≥1 and finally patients with CVD at metastatic disease vs. without CVD.

For all cancer-control outcome estimates, univariable, as well as multivariable Cox regression models were applied. Adjustment in multivariable Cox regression models were performed for Gleason Score (6-7 vs. 8-10), synchronous vs. metachronous mHSPC and high volume mHSPC (vs. low volume). Moreover, depending on the outcome variable of interest, adjustment for age at metastatic disease (<75 vs. ≥75 yrs), ECOG status at metastatic disease (0 vs. 1-2) and CVD (vs. no CVD) was made. For PFS analyses further adjustment for the number of treatment line (1^st^ to 7^th^) and for OS analyses for the number of received CRPC treatment lines was made. All tests were two sided with a level of significance set at p < 0.05. R software environment for statistical computing and graphics (version 3.4.3) was used for all analyses.

## Results

Among the 312 mCRPC patients included in the study (Table 1), the median age at metastatic diagnosis was 70 years (IQR: 62-74 years), with a median PSA level at mCRPC of 16 ng/ml (IQR: 5-60 ng/ml). Overall, 47% of patients were classified as having an ECOG performance status of ≥1, and 35% had active or previously treated cardiovascular disease. At initial diagnosis, 59% presented with de novo metastatic disease, and 43% received some form of local treatment to the prostate. The median number of systemic treatments received for mCRPC was three (IQR: 2-5), while the median number of [^177^Lu]Lu-PSMA therapy cycles was four (IQR: 2-6). The median PSA reduction observed during [^177^Lu]Lu-PSMA therapy was 17% (IQR: 0-62%). At the time of mHSPC diagnosis, 65% of patients had high-volume disease, and 59% were de novo metastatic. At mCRPC stage, the majority (82%) presented with bone-only metastases, while 8.7% and 9.4% had lymph node or visceral metastases (with or without bone involvement), respectively.

### Baseline characteristics: Age group ≤75 vs. >75 years

Of 312 [^177^Lu]Lu-PSMA treated mCRPC patients, 76% (n = 238) were ≤75 years at time of metastatic diagnosis (Table 1), while 24% (n = 74) were aged >75 years. In comparison between both age groups, the median number of received systemic treatment lines (4 vs. 3, p < 0.001) was higher for patients ≤75 years. Patients aged >75 years received [^177^Lu]Lu-PSMA more frequently within the first three systemic lines of mCRPC, compared to patients aged ≤75 years (85% vs. 62%, p < 0.001). Moreover, patients with ECOG status ≥2 were significantly more prevalent in patients aged >75 years (13% vs. 4.3%, p < 0.01). Conversely, no difference was observed regarding median PSA response under [^177^Lu]Lu-PSMA radioligand therapy (-20% vs. -14%) and rate of PSA50 (31% vs. 33%) between patients aged ≤75 vs. >75 years (both p>0.9).

### [^177^Lu]Lu-PSMA PFS and OS analyses stratified according to age

**Figure 1:**
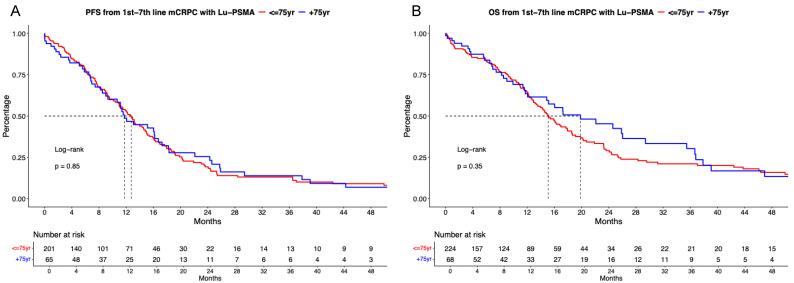
Kaplan Meier curves depicting progression-free survival (PFS, A) and overall survival (B) in first to seventh-line metastatic castration-resistant prostate cancer (mCRPC) patients receiving [^177^Lu] Lutetium prostate-specific membrane antigen (Lu-PSMA) radioligand therapy and stratified according to age at metastatic disease.

In PFS analyses between [^177^Lu]Lu-PSMA treated patients, no significant difference between patients aged ≤75 vs. >75 years was observed (Figure 1A, hazard ratio [HR]: 0.97, p = 0.85) with median PFS of 12.7 vs. 11.7 months. Corresponding 12-months PFS rates were 53.2 vs. 48.7%.

In OS analyses, also no differences between both age categories were observed (Figure 1B, HR: 0.85, p = 0.4) with median OS of 15.1 vs. 19.8 months. Corresponding 12-months OS rates were 64.7 vs. 63.4%. In multivariable Cox regression models, also no significant difference for PFS (Table 2A) and OS (Table 2B) were observed regarding age ≤75 vs. >75-year-old mCRPC patients receiving [^177^Lu]Lu-PSMA (both p≥0.4).

### [^177^Lu]Lu-PSMA PFS and OS analyses stratified according to ECOG status

**Figure 2:**
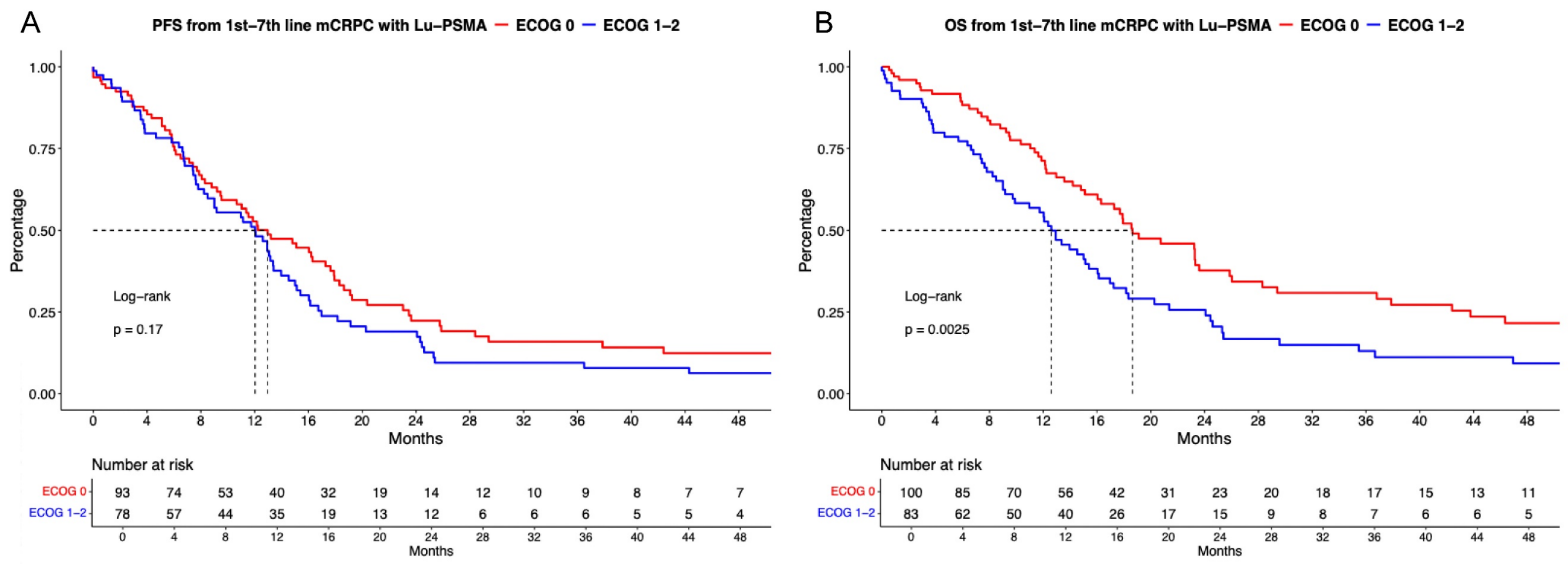
Kaplan Meier curves depicting progression-free survival (PFS, A) and overall survival (B) in first to seventh-line metastatic castration-resistant prostate cancer (mCRPC) patients receiving [^177^Lu] Lutetium prostate-specific membrane antigen (Lu-PSMA) radioligand therapy and stratified according to Easter Cooperative Oncology Group (ECOG) status.

Of [^177^Lu]Lu-PSMA patients with known ECOG status, 54% (n = 105) were categorized as ECOG status 0 vs. 46% (n = 95) ECOG status ≥1. In PFS analyses (Figure 2B), no significant difference between ECOG 0 vs. ≥1 was observed with a HR of 1.26 and median PFS of 13.0 vs. 12.0 months (p = 0.17). Corresponding 12-months PFS rats were 52.7% vs. 51.1% for ECOG 0 vs. ≥1. After multivariable adjustment in Cox regression models (Table 2A), no significant difference was observed (p = 0.4).

In OS analyses (Figure 2B), patients with ECOG 0 harbored significant better OS rates than patients with ECOG ≥1 undergoing [^177^Lu]Lu-PSMA radioligand therapy with a HR of 1.69 (p < 0.01) and median OS of 18.6 vs. 12.6 months. Corresponding 12-months OS rates were 71.2% vs. 55.5% for ECOG 0 vs. ≥1. However, after further multivariable adjustment in Cox regression models, no further differences between ECOG status groups were observed (p = 0.8, Table 2B).

### [^177^Lu]Lu-PSMA PFS and OS analyses stratified according to CVD

**Figure 3:**
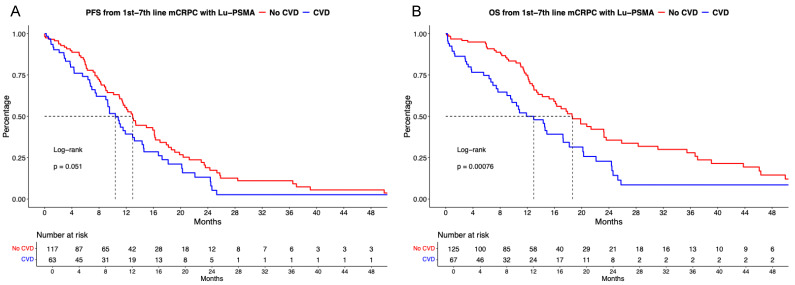
Kaplan Meier curves depicting progression-free survival (PFS, A) and overall survival (B) in first to seventh-line metastatic castration-resistant prostate cancer (mCRPC) patients receiving [^177^Lu] Lutetium prostate-specific membrane antigen (Lu-PSMA) radioligand therapy and stratified according active or history of cardiovascular disease (CVD).

Of [^177^Lu]Lu-PSMA patients with available CVD status, 66% (n = 133) harbored no CVD vs. 34% (n = 70) with active or previous CVD. PFS analyses failed to provide significant differences between CVD vs. no CVD (Figure 3A), with median PFS of 13.0 vs. 10.4 months (HR: 1.44, p = 0.051). Corresponding 12-months PFS rates were 55.4% vs. 39.3% for no CVD vs. CVD groups. After multivariable adjustment, CVD was a significant predictor of shorter PFS (HR: 2.79, p < 0.01, Table 2A).

In OS analyses (Figure 3B), significant OS differences for [^177^Lu]Lu-PSMA patients without CVD vs. CVD was observed (HR: 1.93, p < 0.01) with median OS of 18.6 vs 12.9 months. Corresponding 12-months OS rates were 74.9% vs. 50.0% for no CVD vs. CVD groups. After further multivariable adjustment in Cox regression models, CVD was also an independent predictor for worse OS (HR: 5.14, p < 0.01, Table 2B).

## Discussion

We initially hypothesized that important cancer-control differences may exist in mCRPC patients treated with [^177^Lu]Lu-PSMA and aged >75 years relative to mCRPC patients aged ≤75 years. Moreover, we also hypothesized that additional cancer-control outcome differences may exist in patients classified as ECOG ≥1 status or with history or active CVD. We tested these hypotheses within the FRAMCAP database and made several important observations.

First, we observed that in real-world setting, approximately every fourth patients (24%) receiving [^177^Lu]Lu-PSMA treated is aged >75 years at time of metastatic diagnosis. Moreover, we observed that patients aged above 75 years and receiving [^177^Lu]Lu-PSMA receive in median three systemic treatment lines for mCRPC, which were significantly less than in patients ≤75 years (four systemic treatment lines). Additionally, patients aged >75 years, received [^177^Lu]Lu-PSMA significantly more frequently within the first three treatment lines for mCRPC (85% vs. 62%), which might also contribute to the favorable OS outcomes. All of the above observations are of note, since they reflect a deep adaption of [^177^Lu]Lu-PSMA mCRPC treatment in clinical practice and treatment algorithms, as well as an early administration in elderly patients (even outside current EMA approval). Moreover, these findings are significant, since patients aged >75 years are frequently underrepresented within phase III randomized trials and the focus does not primarily lie on elderly subgroups. For example, in the VISION trial, the basis for approval of [^177^Lu]Lu-PSMA after previous taxan-based chemotherapy and ARPI treatment, enrolled patients with a median age of 70 years, with the youngest patient included aged 48 years 11. Similarly, in the TheraP trial, patients were enrolled at a median age of 72 years, showing a different focus within these studies 15. Compared to previous real-world studies, our cohort of elderly mCRPC patients indicate that [^177^Lu]Lu-PSMA may provide clinicians with a feasibly additional mCRPC treatment option, since median number of received treatment therapies for mCRPC was higher within our study than for example in a report by Fernando *et al.* in which mCRPC patients aged >75 years. In this report, 66% of patients received only one line of treatment 21. Similar observations were also made within other publications, addressing elderly mCRPC patients 22,23.

Second, when cancer-control outcomes of [^177^Lu]Lu-PSMA mCRPC patients aged >75 years at time of metastases were compared, comparable median PFS and OS outcomes were observed and no difference were found after further additional adjustment in Cox regression models. This clearly shows the high effectivity of [^177^Lu]Lu-PSMA also in elderly patients. Moreover, PSA responses were similar between both age groups. These observations are of importance, since no previous published report focused on cancer-control outcomes of elderly [^177^Lu]Lu-PSMA patients. Subgroup analyses of currently available phase III trials such as the VISION or SPLASH trial provide only analyses of patients aged ≥65 years 11,14. A previously presented abstract at ESMO 2024 comparing [^177^Lu]Lu-PSMA in French mCRPC patients pre-treated with at least one taxane-based and at least one APRI-based treatment in patients ≥75 years found also no difference between PFS and OS 24. However, PFS was shorter within these cohort with 7 vs. 8 months for patients aged ≥75 vs. <75 years. These differences may be explained by the heavily pre-treatment of mCRPC patients, while patients within our study also received [^177^Lu]Lu-PSMA in more previous systemic treatment lines of mCRPC and median PFS usually decreased with every additional treatment line 25. Moreover, PFS definitions may differed. However, our data on reported OS rates are similar to previously published real-world reports about [^177^Lu]Lu-PSMA treatment, irrespectively of stratification regarding age 19,26-28. Moreover, in further sensitivity analyses for OS defined from beginning of mCRPC, also no difference in OS was observed for comparison in both age categories (data not shown).

Finally, we further explored our above findings within [^177^Lu]Lu-PSMA mCRPC patients stratified according to ECOG performance status and CVD. Here, we observed that patients with worse ECOG performance status are of higher risk of death under [^177^Lu]Lu-PSMA. However, these OS difference vanished after adjustment for differences in baseline patient and tumor characteristics in multivariable Cox regression models. In CVD analyses, CVD showed to be associated with shorter non-significant PFS (p = 0.051) and significant shorter OS. Moreover, CVD was independently associated with worse PFS and OS even in multivariable adjusted Cox regression models. These observations are of note since previously mCRPC studies reported worse cancer-control outcomes with worse ECOG performance status, but these findings have never been investigated within specific [^177^Lu]Lu-PSMA mCRPC patients 29. Similarly, stratification according to CVD in [^177^Lu]Lu-PSMA has never been addressed. However, a previously published small-sized report (n = 11) of mCRPC patients undergoing [^177^Lu]Lu-PSMA showed no severe cardiotoxicity profile or adverse events 30. Although our study could not differentiate between deaths due to cardiotoxic events and those related to cancer progression, this represents an important area for further investigation. Notably, prospective trials such as VISION and TheraP, as well as real-world data, have not identified significant cardiotoxicity associated with [^177^Lu]Lu-PSMA that would necessitate routine pretreatment cardiac assessments 11,15. The observed worse PFS in patients with CVD might instead be explained by factors such as higher PSA levels in advanced lines of mCRPC and fewer median cycles of [^177^Lu]Lu-PSMA received in this cohort (data not shown). These findings underline the need for future studies to validate the observed associations in prospective settings and to better understand the interplay between preexisting CVD and outcomes in mCRPC patients undergoing [^177^Lu]Lu-PSMA treatment.

In addition to the above-mentioned limitation, our study should be interpreted in its light of the retrospective and single-center design. Moreover, some missing data, limitations in their further distinction (such as different CVD conditions), as well as other not reported variables may have influenced cancer-control outcomes. Further, no information regarding adverse events or blood values other than PSA were available, similar to possible dose reductions or adjustments in treatment schedules. Moreover, in comparison to other trails one has to keep in mind that used [^177^Lu]Lu-PSMA was seen as equivalent to commercially available one. Finally, some of the reported subgroup analyses may lack of sample size and therefore may limit the findings.

Taken together, the current real-world cohort of [^177^Lu]Lu-PSMA mCRPC patients suggest feasible cancer-control outcomes such as PFS and OS for patients aged >75 years. Therefore, [^177^Lu]Lu-PSMA should be considered as a safe and important cornerstone in the treatment of elderly mCRPC patients. Moreover, the study provides important observations of [^177^Lu]Lu-PSMA mCRPC with advanced ECOG performance status and CVD. Especially, patients with CVD should be treated with caution to prevent cardiotoxic events.

## Figures and Tables

**Table 1:** Characteristics of 312 metastatic castration resistant prostate cancer (mCRPC) patients receiving [^177^Lu] Lutetium prostate-specific membrane antigen ([^177^Lu]Lu-PSMA) radioligand therapy stratified according to age at metastatic disease.

Characteristic	N	OverallN = 312*^1^*	Age ≤75, N = 238 (76%)*^1^*	Age >75, N = 74 (24%)*^1^*	p-value*^2^*
Age at metastatic disease, yrs	312	70 (62, 74)	67 (61, 71)	79 (77, 82)	<0.001
PSA at mCRPC, ng/ml	150	16 (5, 60)	16 (4, 46)	21 (7, 91)	0.2
PSA second line mCRPC, ng/ml	181	49 (13, 139)	52 (12, 148)	44 (16, 132)	>0.9
Received systemic treatment lines for mCRPC	312	3 (2, 5)	4 (3, 5)	3 (2, 4)	<0.001
Cycles ^177^Lu-PSMA	267	4 (2, 6)	3 (2, 6)	4 (2, 6)	0.6
PSA response under ^177^Lu-PSMA, %	57	17 (0, 62)	20 (0, 62)	14 (0, 68)	>0.9
PSA response 50%	57	18 (32%)	14 (31%)	4 (33%)	>0.9
PSA response 90%	57	6 (11%)	4 (8.9%)	2 (17%)	0.6
Treatment ^177^Lu-PSMA	308				<0.001
1st line mCRPC		28 (9.1%)	15 (6.4%)	13 (18%)	
2nd line mCRPC		72 (23%)	47 (20%)	25 (34%)	
3rd line mCRPC		108 (35%)	84 (36%)	24 (33%)	
4th line mCRPC		38 (12%)	35 (15%)	3 (4.1%)	
5th line mCRPC		40 (13%)	34 (14%)	6 (8.2%)	
6th line mCRPC		16 (5.2%)	15 (6.4%)	1 (1.4%)	
7th line mCRPC		6 (1.9%)	5 (2.1%)	1 (1.4%)	
ECOG status	186				0.002
0		100 (54%)	85 (61%)	15 (33%)	
1		74 (40%)	49 (35%)	25 (54%)	
≥2		12 (6.5%)	6 (4.3%)	6 (13%)	
CVD	189	66 (35%)	47 (32%)	19 (46%)	0.083
Gleason score 8-10	267	197 (74%)	146 (73%)	51 (77%)	0.5
Local therapy prostate	312	133 (43%)	104 (44%)	29 (39%)	0.5
High volume mHSPC	122	79 (65%)	62 (64%)	17 (68%)	0.7
De Novo mHSPC	307	180 (59%)	146 (62%)	34 (47%)	0.017
Metastatic sites at mCRPC	138				0.14
M1a		12 (8.7%)	7 (6.9%)	5 (14%)	
M1b		113 (82%)	83 (81%)	30 (83%)	
M1c		13 (9.4%)	12 (12%)	1 (2.8%)	
Treatment first-line mCRPC	312				<0.001
ADT mono		26 (8.3%)	25 (11%)	1 (1.4%)	
Chemotherapy		56 (18%)	52 (22%)	4 (5.4%)	
^177^Lu-PSMA		28 (9.0%)	15 (6.3%)	13 (18%)	
ARPI		176 (56%)	125 (53%)	51 (69%)	
PARPi		1 (0.3%)	1 (0.4%)	0 (0%)	
Radium		24 (7.7%)	19 (8.0%)	5 (6.8%)	
None/Other/NA		1 (0.3%)	1 (0.4%)	0 (0%)	
*^1^* Median (Q1, Q3); n (%)					
*^2^* Kruskal-Wallis rank sum test; Fisher's exact test; Pearson's Chi-square test					

Abbreviations: PSA: Prostate-specific antigen, ECOG: Eastern Cooperative Oncology group, CVD: Cardiovascular disease, mHSPC: metastatic hormone-sensitive prostate cancer, ADT: Androgen deprivation therapy, ARPI: Androgen receptor pathway inhibitor, nmHSPC: non-metastatic hormone-sensitive prostate cancer, m0CRPC: non-metastatic CRPC, PARPi: Poly adenosine diphosphate ribose polymerase inhibitor

**Table 2:** Univariable und multivariable Cox regression models predicting progression-free survival (PFS; A) and overall survival (OS; B) in metastatic castration resistant prostate cancer (mCRPC) patients receiving [^177^Lu] Lutetium prostate-specific membrane antigen ([^177^Lu]Lu-PSMA) radioligand therapy. Abbreviation: HR: Hazard Ratio, CI: Confidence interval, ECOG: Eastern Cooperative Oncology Group, CVD: Cardiovascular disease

	Univariable			Multivariable		
PFS	**HR**	**CI**	**p value**	**HR**	**CI**	**p value**
Age ≤75 yrs	**Ref.**	**-**	**-**	**Ref.**	**-**	**-**
Age >75 yrs	0.97	0.70-1.34	0.9	0.62	0.21-1.88	0.4
ECOG 0	**Ref.**	**-**	**-**	**Ref.**	**-**	**-**
ECOG 1-2	1.26	0.91-1.77	0.17	0.68	0.29-1.64	0.4
No CVD	**Ref.**	**-**	**-**	**Ref.**	**-**	**-**
CVD	1.44	1.00-2.07	0.051	2.79	1.29-6.00	<0.01
OS	**HR**	**CI**	**p value**	**HR**	**CI**	**p value**
Age ≤75 yrs	**Ref.**	**-**	**-**	**Ref.**	**-**	**-**
Age >75 yrs	0.85	0.60-1.20	0.4	0.98	0.25-3.91	>0.9
ECOG 0	**Ref.**	**-**	**-**	**Ref.**	**-**	**-**
ECOG 1-2	1.69	1.20-2.39	<0.01	0.89	0.34-2.36	0.8
No CVD	**Ref.**	**-**	**-**	**Ref.**	**-**	**-**
CVD	1.93	1.31-2.84	<0.01	5.14	2.10-12.60	<0.01

Adjustment in multivariable Cox regression models for: Gleason Score (6-7 vs. 8-10), synchronous vs. metachronous mHSPC, high volume mHSPC (vs. low volume). Depending on variable of interest, further adjustment for age (<75 vs. ≥75 yrs), ECOG status (0 vs. 1-2) and CVD (vs. no CVD) was made. For PFS further adjustment for number of treatment line (1^st^ to 7^th^) and for OS number of received CRPC treatment lines was made.
